# Meibomian gland alterations in allergic conjunctivitis: insights from a novel quantitative analysis algorithm

**DOI:** 10.3389/fcell.2024.1518154

**Published:** 2025-01-06

**Authors:** Jingting Wei, Kunhong Xiao, Qingyuan Cai, Shenghua Lin, Xiangjie Lin, Yujie Wang, Jiawen Lin, Kunfeng Lai, Yunxi Ye, Yuhan Liu, Li Li

**Affiliations:** ^1^ Fujian Provincial Hospital, Shengli Clinical Medical College of Fujian Medical University, Fuzhou University Affiliated Provincial Hospital, Fuzhou, China; ^2^ Department of Optometry, School of Medical Technology and Engineering, Fujian Medical University, Fuzhou, China; ^3^ Centre for Eye Research Australia, Royal Victorian Eye and Ear Hospital, Melbourne, VIC, Australia; ^4^ Division of Ophthalmology, Department of Surgery, University of Melbourne, Melbourne, VIC, Australia; ^5^ School of Computer Science and Big Data, Fuzhou University, Fuzhou, China; ^6^ School of Basic Medical Sciences, Fujian Medical University, Fuzhou, China

**Keywords:** allergic conjunctivitis, meibomian gland, quantitative analysis algorithm, dry eye disease, artificial intelligence

## Abstract

**Purpose:**

To investigate the changes in meibomian gland (MG) structure in allergic conjunctivitis (AC) patients using an intelligent quantitative analysis algorithm and to explore the relationship between these changes and clinical parameters.

**Methods:**

A total of 252 eyes from patients with AC and 200 eyes from normal controls were examined. Infrared meibography was performed using the non-contact mode of the Keratograph 5M. MG images were analyzed using a deep learning-based a quantitative analysis algorithm to evaluate gland length, area, dropout ratio, and deformation. Clinical parameters, including tear meniscus height, tear break up time (TBUT), conjunctival hyperemia, and Ocular Surface Disease Index (OSDI) scores, were assessed and correlated with changes in the structure of MG.

**Results:**

The average MG length in AC patients was 4.48 ± 1.04 mm, shorter compared to the control group (4.72 ± 0.94 mm). The average length of the central 5 glands in AC patients was 4.94 ± 1.67 mm, which was also shorter than the control group’s central 5 glands (5.38 ± 1.42 mm). Furthermore, the central 5 glands’ area in AC patients (1.61 ± 0.64 mm^2^) was reduced compared to the control group (1.79 ± 0.62 mm^2^). Tear meniscus height was lower in the allergy group (0.26 ± 0.10 mm) compared to the control group (0.44 ± 0.08 mm) (P < 0.05). The non-invasive first tear film break-up time was shorter in the allergy group (8.65 ± 6.31 s) than in the control group (10.48 ± 2.58 s) (P < 0.05). Conjunctival congestion was higher in the allergy group (1.1 ± 0.52) compared to the control group (0.97 ± 0.30) (P < 0.05). The OSDI score in the allergy group (8.33 ± 7.6) was higher than that in the control group (4.00 ± 0.50) (P < 0.05). Correlation analysis revealed that the gland dropout ratio was positively associated with male gender and negatively associated with age and OSDI scores. Additionally, despite an increased number of MG, tear film stability was not improved.

**Conclusion:**

Through the intelligent quantitative algorithm, we found that AC leads to significant changes in MG structure, particularly affecting gland length and central area.

## 1 Introduction

Over recent decades, the incidence of allergic conjunctivitis (AC) has steadily increased, with prevalence estimates ranging from 6% to 30% in the general population (Leonardi et al., 2015). AC is characterized by symptoms such as itching, redness, a whitish conjunctiva, and sometimes thick mucous discharge, severe cases may present with papillary hypertrophy of the palpebral conjunctiva (Friedlaender, 1993). Notably, AC is recognized as a key risk factor for inducing dry eye disease (DED) (Villani et al., 2018), significantly impacting patients’ quality of life. The meibomian glands (MGs), located within the eyelids, play a crucial role in maintaining the stability of the tear film by secreting its lipid layer, thereby reducing tear evaporation (Dietrich et al., 2021). Recent studies have shown that AC can contribute to meibomian gland dysfunction (MGD) (Mizoguchi et al., 2017a), leading to tear film instability and exacerbation of ocular surface discomfort.

Previous research has highlighted the potential impact of allergic conjunctivitis on MG morphology. Arita et al. (2012) suggested that allergic reactions might be the reason for increased MG deformation in patients with contact lens-associated allergic conjunctivitis. Similarly, Liu et al. (2022) proposed that morphological and cytological changes in the MGs were more pronounced in patients with seasonal allergic conjunctivitis. Moreover, Wu et al. found that these relationships differed between adults and children (Wu et al., 2022a). Additionally, A 1-unit increase in PM2.5 levels is associated with a 0.06 increase in ocular inflammation and a 0.07 increase in gland dropout (Huang et al., 2020). This suggests that PM2.5 may damage the glands by inducing inflammation. However, most existing studies rely heavily on subjective assessment methods, such as manual analysis and qualitative classification, which compromise the reliability and reproducibility of their findings. These studies often fail to comprehensively capture the patterns of MG morphological changes across different populations.

With the advent of intelligent algorithms, the automated quantitative analysis of MG images has garnered increasing attention from researchers (Yeh et al., 2021; Xiao et al., 2021; Li et al., 2024; Yang et al., 2023; Dai et al., 2021). Deep learning-based segmentation methods for MG have been developed (Saha et al., 2022; Lin et al., 2024; Wu et al., 2024), allowing automated quantitative analysis of various MG parameters, such as length, width, and tortuosity, the latter referring to the twisting or winding nature of the glands, by learning features from large, unlabeled meibography datasets (Setu et al., 2021). AI-based quantitative analysis algorithms offer more objective and precise assessments of MG parameters, providing new tools and perspectives for studying changes in MGs associated with AC.

This study aims to utilize an intelligent quantitative analysis algorithm to systematically evaluate the microstructural changes of MGs in patients with AC, exploring the relationships between these changes and clinical relevant indicators, such as OSDI scores and TBUT. This study incorporates intelligent analysis technology to provide a more objective and accurate assessment of MGs than traditional methods to offer deeper insights into the diagnosis and management of AC.

## 2 Materials and methods

### 2.1 Study subjects and grouping

**FIGURE 1:**
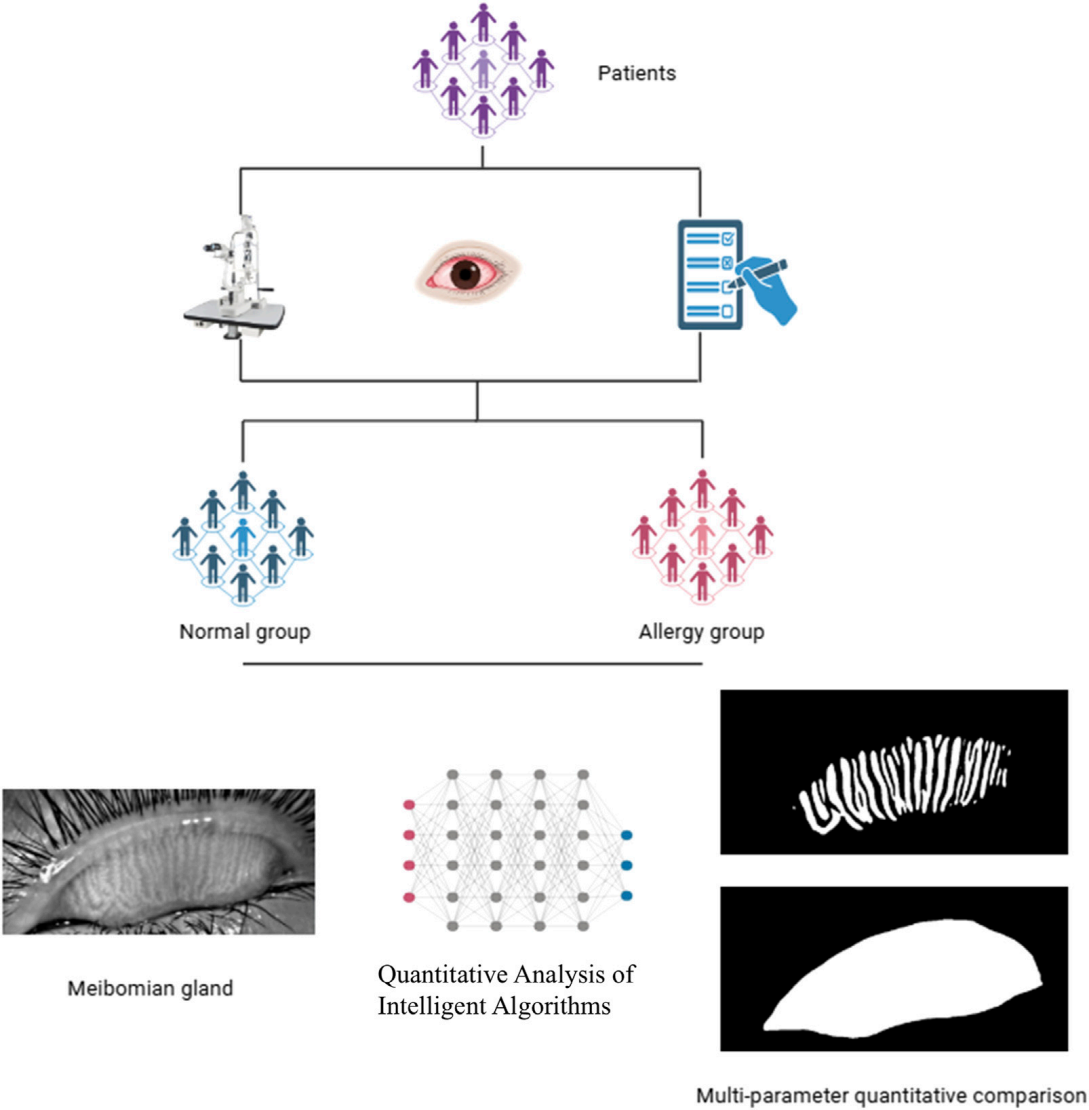
Study flowchart: MG analysis workflow based on AI algorithm.

This study collected data from patients who visited the Department of Ophthalmology at Fujian Provincial Hospital between January 2024 and October 2024, and the research was conducted following a defined protocol (Figure 1). We performed a power analysis to determine the required sample size. Based on an expected effect size (Cohen’s d = 0.5) and a significance level (α = 0.05), the power analysis indicated that a minimum of 64 eyes per group is needed to achieve 80% power. The inclusion criteria consisted of patients clinically diagnosed with AC through a comprehensive examination. According to the diagnosis, AC patients exhibited typical symptoms such as ocular itching, conjunctival hyperemia, and tearing. A total of 252 eyes were included in the AC group, while 200 eyes without AC were assigned to the control group. The allergic group had an average age of 11.78 ± 6.77 years, with 153 males and 99 females, whereas the control group had an average age of 11.65 ± 9 years, including 121 males and 79 females. The exclusion criteria were as follows: a) patients unable to cooperate with the examination; b) patients with other organic eye diseases; c) patients with a history of ocular surgery; d) patients with ocular or systemic conditions that could interfere with tear film production or function.

This study was approved by the Ethics Committee of Fujian Provincial Hospital (K202410006), and all procedures strictly adhered to the ethical principles of the Declaration of Helsinki. Prior to the study, all participants signed informed consent forms, and all clinical examinations were performed by experienced ophthalmologists.

### 2.2 Clinical examinations and data collection

Clinical examinations were conducted with careful adjustments to the height of the patient’s chair and examination table, ensuring optimal comfort for the patient. During the examination, the patient’s chin was positioned on the chin rest, and the forehead was secured against the forehead support, allowing them to focus on the center of the examination instrument.

#### 2.2.1 Data collection involved several key metrics

##### 2.2.1.1 Tear meniscus height

Measured using the non-contact infrared imaging system of the Keratograph 5M, this metric assesses the volume of tear film present in the conjunctival sac.

##### 2.2.1.2 Non-invasive first TBUT

After the patient blinked twice and maintained a wide-eyed position, the initial TBUT was automatically calculated by the software. This parameter reflects the stability of the tear film.

##### 2.2.1.3 Non-invasive average TBUT

This metric provides an average of multiple TBUT measurements, offering a more comprehensive evaluation of tear film stability.

##### 2.2.1.4 Conjunctival congestion

This was assessed qualitatively during the examination to determine the extent of vascular engorgement in the conjunctiva.

##### 2.2.1.5 OSDI

The OSDI questionnaire was employed to evaluate ocular surface symptoms and the severity of dry eye disease. Scores range from 0 to 100, with higher scores indicating greater severity of symptoms.

### 2.3 Intelligent quantitative analysis of blepharoplasty images

The intelligent quantitative analysis algorithm for MGs introduces the U-Net++ network architecture to build an automatic MG segmentation model (Lin et al., 2021), enabling deeper exploration of MG morphology in patients with AC.

We imported a total of 452 previously collected infrared blepharoplasty images into the Intelligent Quantitative Analysis System to segment the MG images. The MG segmentation process consists of two stages: training and segmentation, including modules such as data augmentation and gland segmentation (Li et al., 2023). The data augmentation module randomly selects N types from various augmentation methods, including cropping, flipping, shearing, translating, rotating, equalizing, contrast variation, and brightness variation, with random amplitude M for each type. Based on experiments, this study set N to 2 and M to a range of 1–10. This data augmentation module provides more data samples, enhancing the generalizability of the model. The gland segmentation module utilizes the U-Net model, first proposed in 2015, specifically for medical image segmentation. It uses skip connections to fuse shallow and deep semantic feature maps, overcoming information loss during down-sampling and improving segmentation accuracy. U-Net++ is an improvement on U-Net, redesigning the skip connections to improve inference speed (Zhou et al., 2020).

The intelligent MG analysis algorithm involves three steps (Leonardi et al., 2015): dividing the meibography image into different regions (Friedlaender, 1993); segmenting and identifying glands within the regions (Villani et al., 2018); performing multi-parameter quantitative analysis, calculating deformation and tortuosity (Xiao et al., 2021). The deformation coefficient was calculated as follows:

$$\frac{p_{a}\times p_{b}}{length{\left(central\right)}^{2}}\times \left(\frac{\sqrt{\sum_{i=1}^{n}\;{\left(w_{i}-w_{avg}\right)}^{2}}}{n}+1\right)$$

The formula for calculating the deformation coefficient of the gland was developed and refined based on the arc-string ratio model. Here, p_a_ represents the length of the left side of the gland, p_b_ refers to the length of the right side, w_i_ denotes the diameter of the gland measured at each step, and w_avg_ is the average diameter of the gland. The term “length (central)” indicates the length of the central line, while n represents the number of diameters measured. The formula’s minimum value is 1, and the deformation coefficients are dimensionless.

The average gland length, average gland width, average gland area, gland dropout ratio, average gland deformation coefficient and gland count were collected for each image by quantitative analysis of MG images. We also collected the central MG length, central MG width, central MG area, central MG deformation coefficient, and calculated the mean values of each parameter for the 5 MG of the central Region. For each patient, we determined the central 5 MGs based on the total number of glands. If there are n glands in total: When n is an odd number, (n-5)/2 glands are excluded from both the nasal and temporal sides to ensure that the remaining five glands are in the central region. When n is an even number, (n-6)/2 glands are excluded from the nasal side and (n-4)/2 glands from the temporal side to ensure that the remaining five glands are in the central region.

In clinical work, the patient’s glandular condition is assessed predominantly in the central 5–8 glands, and the glands in the central region of the MGs are the clearest, with the average length of the central glands and the percentage of glands being representative of the patient’s major glandular function, diameter may partially reflect the degree of glandular obstruction.

### 2.4 Statistical analysis methods

Statistical analysis was conducted using GraphPad Prism 9.0. Normality tests were performed on continuous variables. Normally distributed data are presented as mean ± standard deviation (Mean ± SD), with group comparisons via independent sample t-tests. Non-normally distributed data are reported as medians (interquartile range), using the Mann-Whitney U test for comparisons. Categorical variables are expressed as frequencies and percentages, analyzed using the Chi-square test or Fisher’s exact test. Two-sided tests assessed differences in clinical examination results (tear meniscus height, non-invasive first TBUT, non-invasive average TBUT, conjunctival hyperemia, and OSDI scores) between the AC and control groups, with significance set at p < 0.05.

## 3 Result

### 3.1 Demographic characteristics and clinical data on two groups

Table 1 shows that the demographic characteristics of the patients in the allergic and control groups. The allergic group had an average age of 11.78 ± 6.77 years ranging from 5 to 77 years, with 153 males and 99 females, whereas the control group had an average age of 11.65 ± 9 years with a range of 7–58, including 121 males and 79 females. Tear meniscus height was lower in the allergy group (0.26 ± 0.10 mm) compared to the control group (0.44 ± 0.08 mm). The non-invasive first tear film break-up time was shorter in the allergy group (8.65 ± 6.31 s) than in the control group (10.48 ± 2.58 s). Conjunctival congestion was higher in the allergy group (1.1 ± 0.52) compared to the control group (0.97 ± 0.30). Additionally, the OSDI score in the allergy group (8.33 ± 7.6) was higher than that in the control group (4.00 ± 0.50).

**TABLE 1:** Demographic characteristics and clinical data of the participants.

Characterizes	Allergy group	Control group	*p*-Value
Age (years)	11.78 ± 6.77	11.65 ± 9	>0.05
Age distribution range (years)	5–77	7–58	
Sex			>0.05
Male	153	121	
Female	99	79	
Tear meniscus height (mm)	0.26 ± 0.10	0.44 ± 0.08	<0.0001
Non-invasive first TBUT (s)	8.65 ± 6.31	10.48 ± 2.58	0.0002
Non-invasive average TBUT (s)	10.53 ± 6.26	11.56 ± 3.67	0.2366
Conjunctival congestion	1.1 ± 0.52	0.97 ± 0.30	0.0116
OSDI	8.33 ± 7.6	4.00 ± 0.50	<0.0001

### 3.2 Comparison of overall MG parameters between the two groups

**FIGURE 2:**
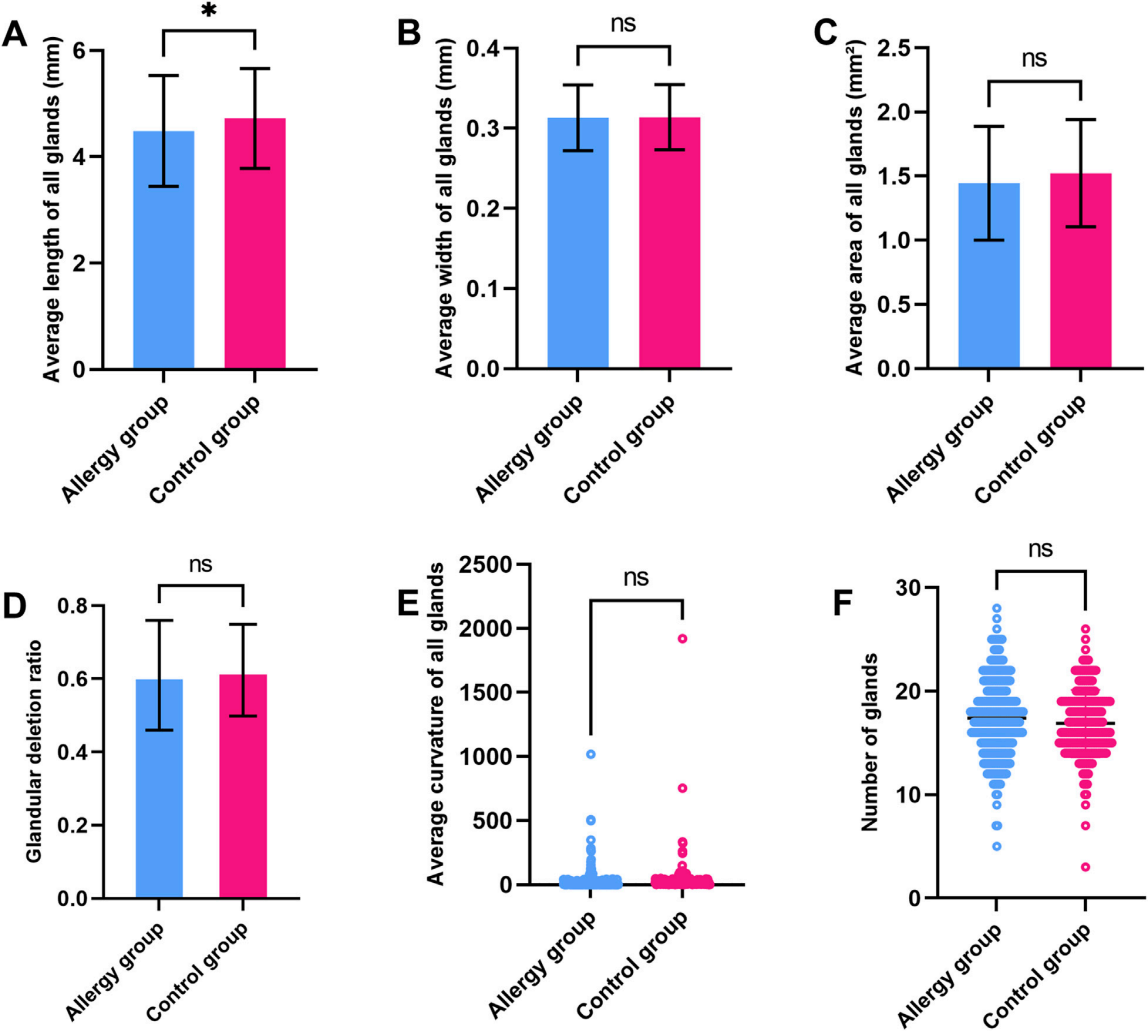
Comparison of MG parameters between the AC and control groups **(A)**. Average gland length **(B)**. Average gland width **(C)**. Average gland area **(D)**. Gland dropout ratio **(E)**. Average Gland Deformation Coefficient **(F)**. Gland count. * indicates a statistically significant difference (p < 0.05); ns indicates no significant difference.

**FIGURE 3:**
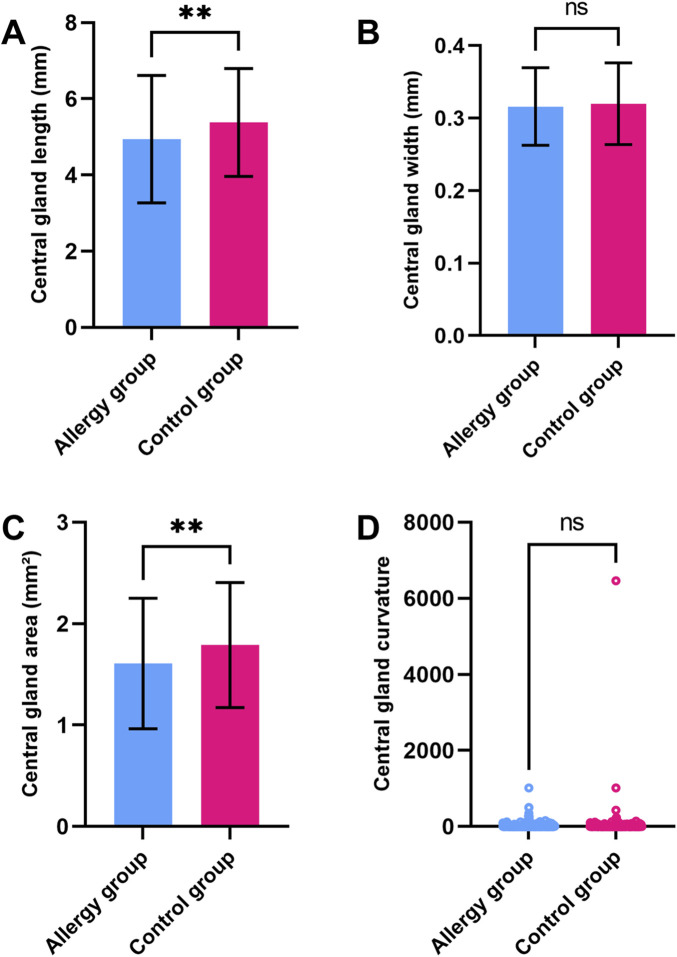
Comparison of central five MG parameters between the AC and control groups **(A)**. central MG length **(B)**. Central MG width **(C)**. Central MG area **(D)**. Central MG deformation coefficient. ** indicates a statistically significant difference (p < 0.01); ns indicates no significant difference.

The average gland length in the control group was significantly longer than in the AC group. However, other parameters such as average gland width, gland area, gland dropout ratio, and gland deformation co-efficient showed no significant differences between the two groups (Figure 2). In the comparison of the central five glands, the length and area of the central MGs in AC group were significantly reduced, while other morphological characteristics showed minimal changes (Figure 3). To further explore potential differences in meibomian gland parameters and other clinical factors, we performed subgroup analyses based on gender. Male and female participants in the allergic and control groups were analyzed separately. In the male subgroup (Supplementary Figures 1, 2), the central MG length and area were significantly higher compared to the AC group. In the female subgroup (Supplementary Figures 3, 4), the average MG length in the control group was greater than that in the AC group. Additionally, the central MG length and area were also significantly larger in the control group compared to the AC group.

### 3.3 Correlation between MG parameters and clinical symptoms in allergic group

**FIGURE 4:**
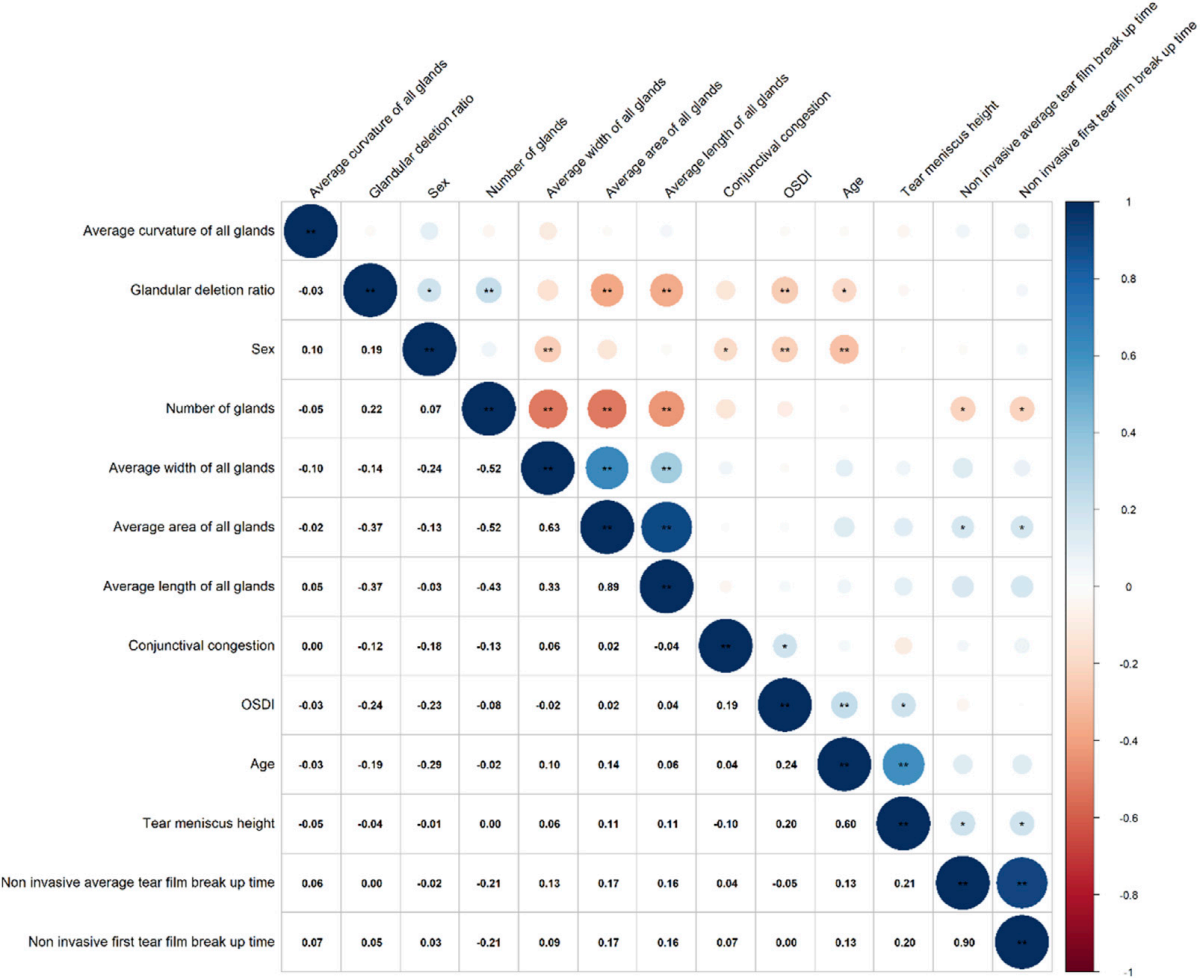
Correlation analysis between MG parameters and clinical symptoms in allergic group.

Correlation analysis (Figure 4) revealed that the gland drop out ratio was positively correlated with gender (0.19) and negatively correlated with OSDI (−0.24) and age (−0.19). Gland count was negatively correlated with non-invasive first TBUT (−0.21) and non-invasive average TBUT (−0.21). Gland area was positively correlated with non-invasive first TBUT (0.17) and non-invasive average TBUT (0.17). Additionally, conjunctival hyperemia was positively correlated with OSDI (0.19).

## 4 Discussion

Although previous studies have identified allergies as a potential risk factor for MG damage (Mizoguchi et al., 2017b; Chao and Tong, 2018; Wu et al., 2022b), these investigations have often been constrained by subjectivity and limited reproducibility. Recent advancements in AI have demonstrated high accuracy and reliability in evaluation of MG parameters (Xiao et al., 2021; Fasanella et al., 2016; Zhang et al., 2022). In this study, we utilized an AI-based quantitative analysis system to systematically examine the changes in MGs in patients with AC, aiming to explore the specific effects of AC on MGs and their correlation with clinically relevant parameters.

Our study found that compared to the control group, the AC group exhibited reduced tear meniscus height, shorter non-invasive first TBUT, increased conjunctival hyperemia, and significantly higher OSDI scores. The reduction in tear meniscus height may reflect decreased tear production or increased tear evaporation, leading to reduced tear retention. The shortened non-invasive first TBUT indicates decreased tear film stability, making the ocular surface more susceptible to external stimuli, causing discomfort. Additionally, the increased conjunctival hyperemia and elevated OSDI scores further confirm the exacerbation of ocular discomfort in AC patients. These findings are consistent with previous studies (Kim and Moon, 2013; Akasaki et al., 2022), which showed that dry eye symptoms are more severe in patients with AC, and the more allergens present, the worse the dry eye symptoms become.

In terms of MG morphology, the overall gland length in the AC group was shorter, and the central five glands showed significantly reduced length and area compared to the control group. This finding further supports the notion that AC affects MG structure, particularly in the central glands, which may be related to chronic inflammation of the ocular surface. The reduction in central gland length and area may result from glandular atrophy or dysfunction caused by local inflammatory responses. Studies have shown that chronic inflammation can trigger immune cells to release various inflammatory mediators, such as cytokines and chemokines, as well as bacterial imbalances, releasing endotoxins, leading to MG structure damage (Mizoguchi et al., 2017b). In our study, there were no significant differences in the width and tortuosity of the central 5 glands, which may be due to the fact that AC-induced inflammation is more likely to damage the openings or ends of the MGs and has less effect on the width and direction of growth of the MGs; and the direction of growth of the MGs is constrained by the anatomical structure of the eyelids, which also allows for the consistency of the overall tortuosity of the MGs. Additionally, mechanical stimulation of the eyes, such as frequent eye rubbing or changes in blinking patterns, may accelerate MG damage, causing glandular atrophy or dysfunction (Wang et al., 2018; Arita et al., 2010).

In the correlation analysis, the gland drop out ratio has a positive correlation of 0.19 with gender, suggesting that male patients may be more prone to gland dropout. This finding aligns with a recent meta-analysis (Hassanzadeh et al., 2021), which revealed that men are more likely to develop MGD, with an estimated overall prevalence of 35.8%. The reasons may be related to different lifestyle habits of men and women, such as smoking and drinking, which can cause some damage to the glands. In addition, it appears that androgens have an effect on gland shortening. The observed negative correlation of −0.24 between gland dropout ratio and OSDI contradicts previous findings, which may be explained by the fact that in allergic conjunctivitis, patient awareness is influenced by various factors, leading to a potential masking of the subjective perception of symptoms. Furthermore, the negative correlation between gland dropout ratio and age may indicate that as children grow older, their gland function gradually develops and matures, leading to a reduction in gland dropout.

Among patients with allergic conjunctivitis, gland count has a negative correlation of −0.21 with both the non-invasive first TBUT and non-invasive average TBUT, this suggests that an increased number of glands can lead to tear film damage in AC patients. The main reason to consider is still because, an increased number of glands may result in loss of epithelial cells and cup cells due to inflammation, produce abnormal or poor-quality lipids, imbalance the tear composition exacerbating tear film breakup (McCulley and Shine, 2004; Nelson et al., 2011). Gland area has a positive correlation of 0.17 with tear breakup time, which is consistent with previous research, suggesting that a more uniform and complete lipid layer helps to cover the tear film surface, reducing tear evaporation and maintaining tear film stability (Mizoguchi et al., 2017c). Conjunctival hyperemia has a positive correlation of 0.19 with OSDI, indicating that the inflammatory response in allergic conjunctivitis directly affects patients’ subjective symptoms, particularly worsening dry eye symptoms.

A key clinical value of our study lies in the use of AI algorithms to assess changes in the meibomian glands of AC patients, which enhances the precision, objectivity, and diversity of parameters. AI algorithms are capable of detecting even the smallest changes, which is especially useful in AC, where the meibomian glands may undergo varying degrees of inflammation or functional damage. Traditional methods often struggle to quantify these subtle pathological changes accurately. Furthermore, AI provides a quantitative evaluation of key meibomian gland parameters, such as gland length, width, area, counts and gland deformation. Additionally, by standardizing the evaluation process, AI algorithms significantly reduce human biases, resulting in more consistent and reliable assessments. These offers a powerful tool for tracking and monitoring the ocular surface conditions of AC patients during follow-up visits.

There are several limitations to this study. First, the analysis is restricted to parameters from the upper eyelid, as previous studies have shown that the upper eyelid provides clearer and higher-quality meibography images compared to the lower eyelid, minimizing uneven focus and demonstrating stronger correlations with clinical indicators (Deng et al., 2021; Daniel et al., 2019). Second, Further research is required to explore the distinct changes in the MGs across these different AC subtypes. Third, various environmental exposures could have also played a role in the results. Factors such as air pollution, climate conditions (e.g., humidity and temperature), and prolonged screen time are known to affect ocular health and meibomian gland function. Future studies should control for these confounding variables. Lastly, the cross-sectional design of this study limits the ability to establish causal relationships. Future longitudinal studies could provide more robust evidence by tracking changes in MG parameters and clinical symptoms over time in a cohort of AC patients.

## 5 Conclusion

This study systematically evaluated changes in the MGs of patients with AC using a novel quantitative analysis algorithm. The findings demonstrate that AC significantly affect the overall length of the MGs, as well as the length and area of the central five glands. These structural changes are strongly associated with decreased tear film stability, increased conjunctival hyperemia, and worsening ocular discomfort. These results underscore the detrimental impact of AC on MG function and overall ocular surface health.

## Data Availability

The original contributions presented in the study are included in the article/Supplementary Material, further inquiries can be directed to the corresponding author.
