# Supplementary material for: Meibomian gland alterations in allergic conjunctivitis: insights from a novel quantitative analysis algorithm
Source: Front Cell Dev Biol. 2025 Jan 6;12:1518154. doi: 10.3389/fcell.2024.1518154 (PMC11743466; doi:10.3389/fcell.2024.1518154)
Supplement: Supplementary file 1 [file Image1.pdf]

Supplementary Materials

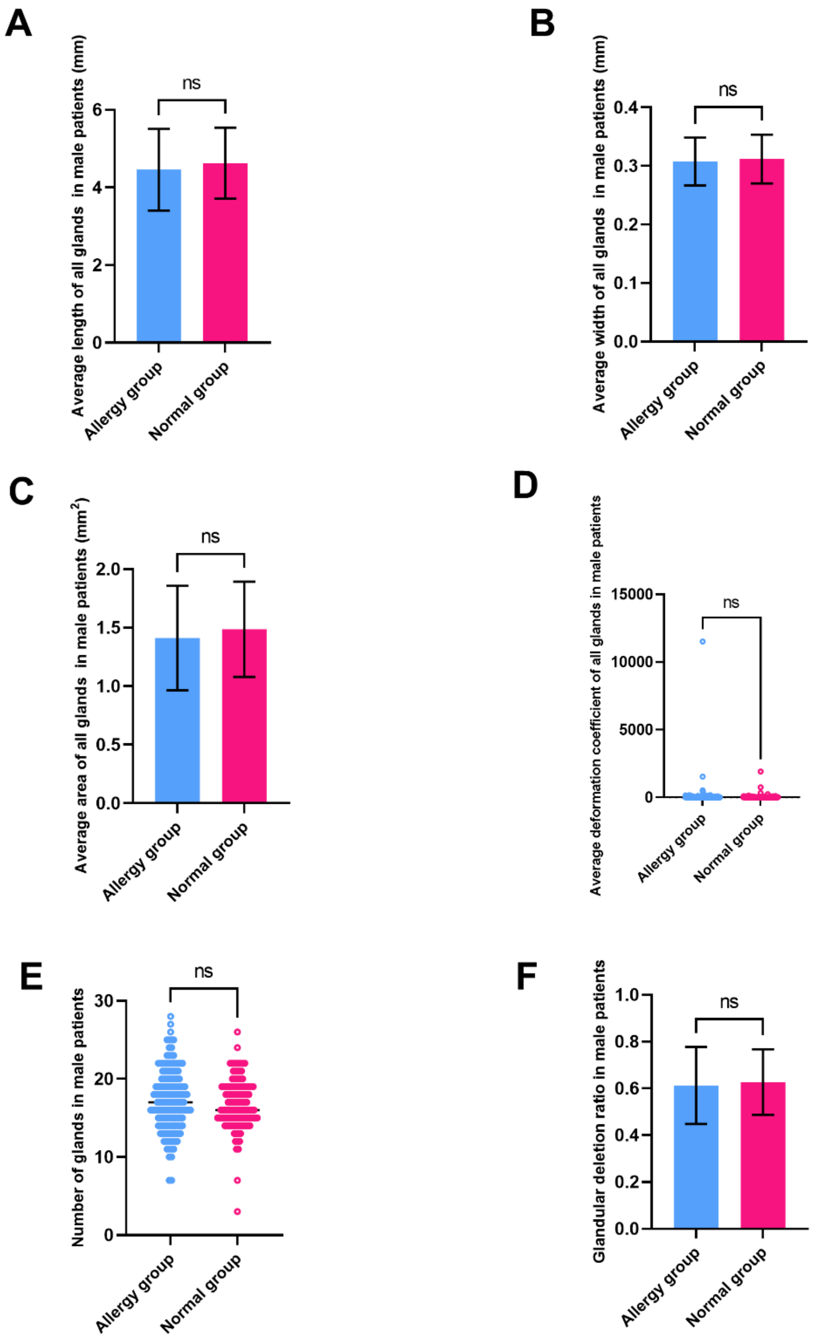

Supplementary Figure 1. Comparison of MG Parameters Between the AC and Control Groups in Males A. Average Gland Length; B. Average Gland Width; C. Average Gland Area; D. Average Gland Deformation Coefficient; E. Gland Count; F. Gland Dropout Ratio. ns indicates no significant difference.

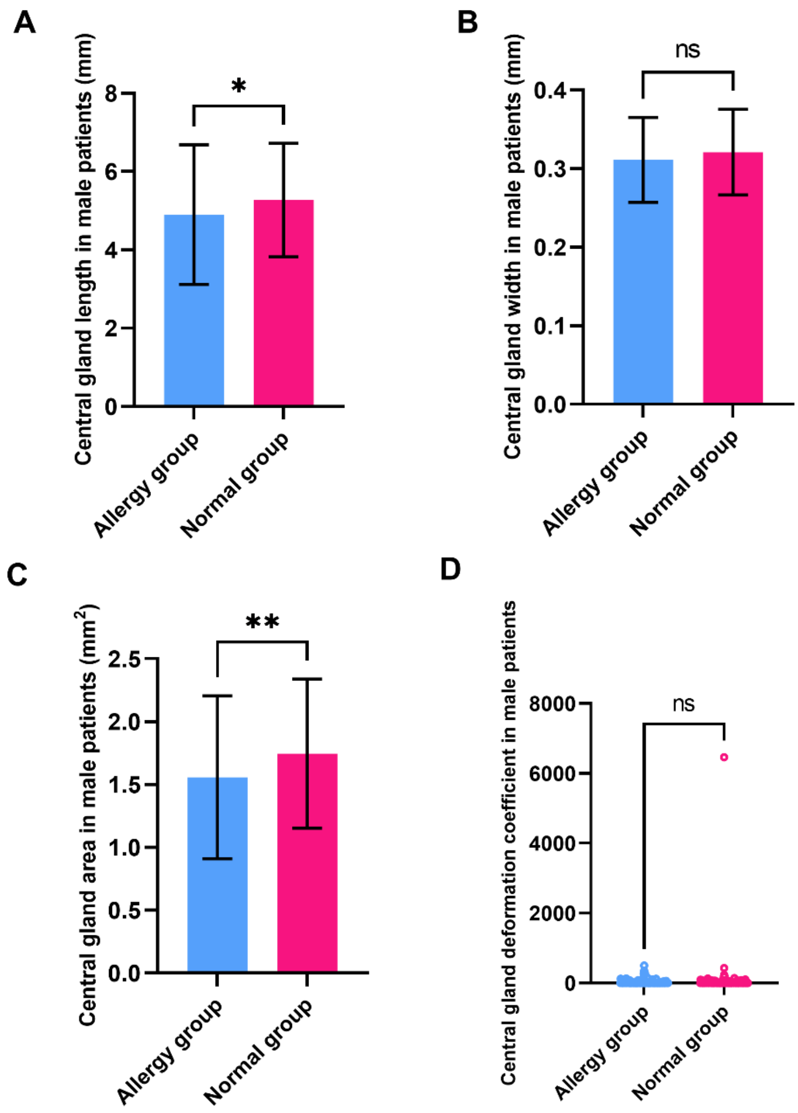

Supplementary Figure 2. Comparison of Central Five MG Parameters Between the AC and Control Groups in Males. A. Central MG Length; B. Central MG Width; C. Central MG Area; D. Central MG Deformation Coefficient. ** indicates a statistically significant difference ($p < 0.01$); ns indicates no significant difference.

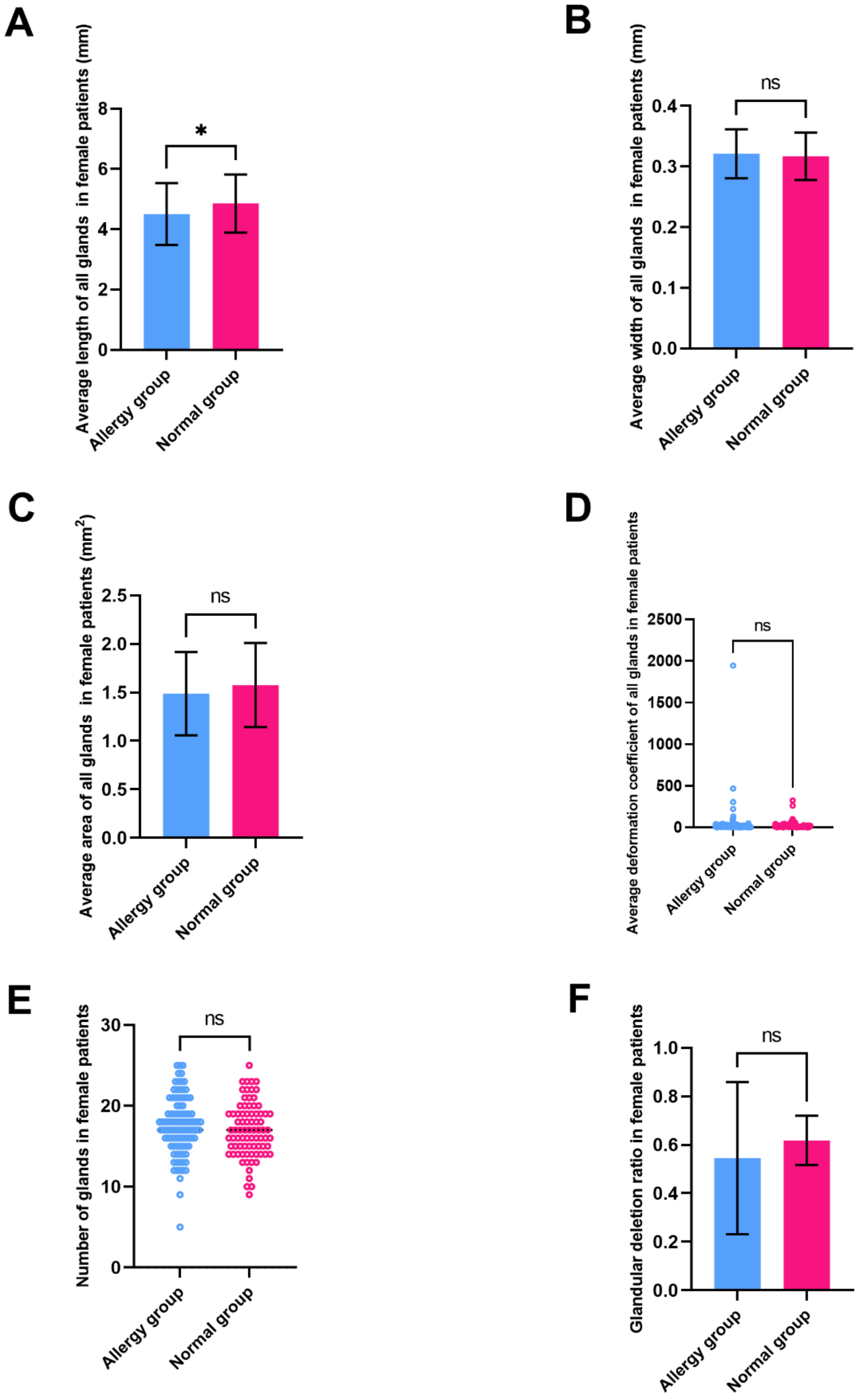

Supplementary Figure 3. Comparison of MG Parameters Between the AC and Control Groups in Females. A. Average Gland Length; B. Average Gland Width; C. Average Gland Area; D. Average Gland Deformation Coefficient; E. Gland Count; F. Gland Dropout Ratio ns indicates no significant difference.

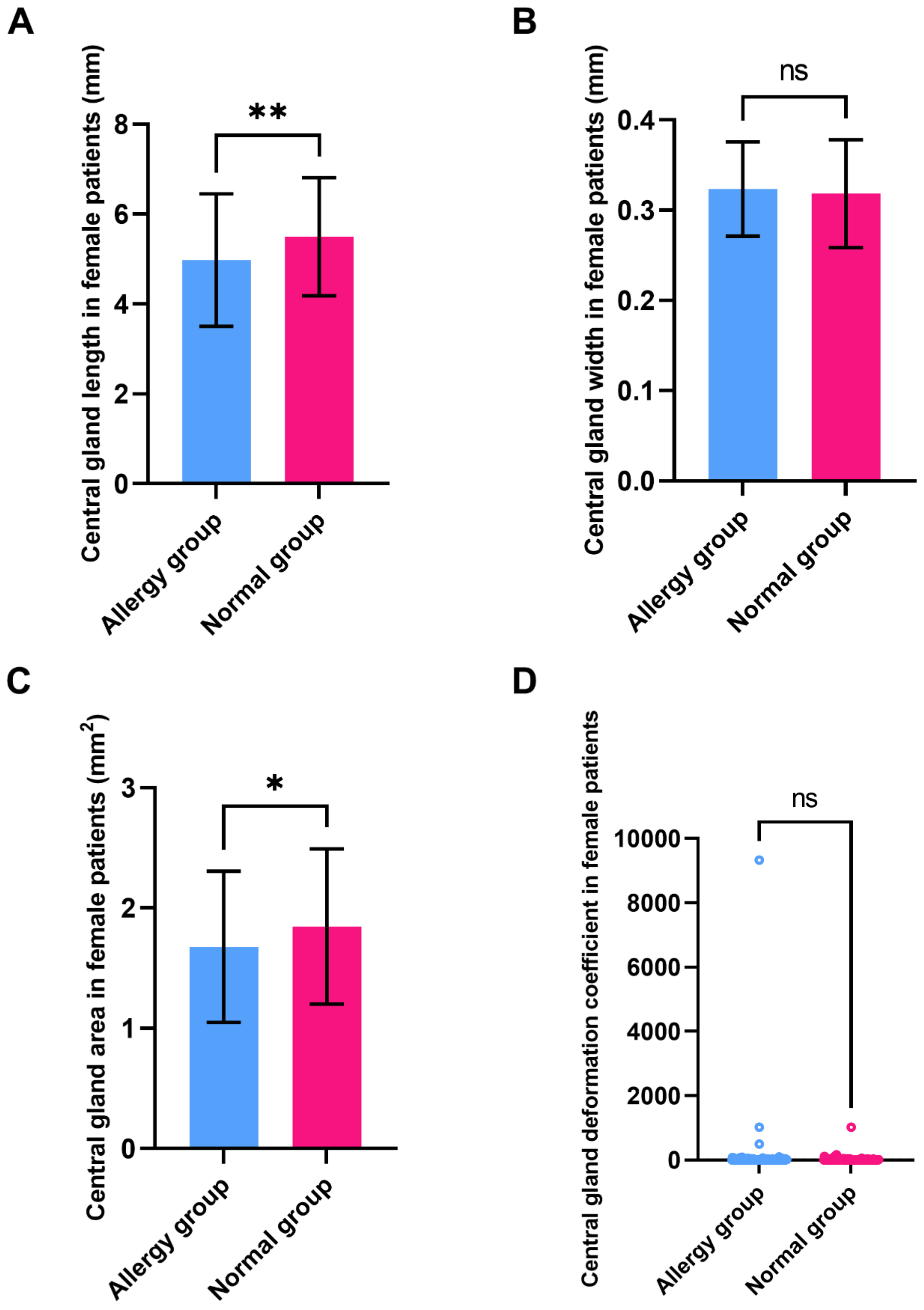

Supplementary Figure 4. Comparison of Central Five MG Parameters Between the AC and Control Groups in Females. A. Central MG Length; B. Central MG Width; C. Central MG Area; D. Central MG Deformation Coefficient. ** indicates a statistically significant difference ($p < 0.01$); ns indicates no significant difference.
